# Targeted for destruction: degradation of singlet oxygen-damaged chloroplasts

**DOI:** 10.1080/15592324.2022.2084955

**Published:** 2022-06-08

**Authors:** Matthew D. Lemke, Jesse D. Woodson

**Affiliations:** The School of Plant Sciences, University of Arizona, Tucson, AZ, USA

**Keywords:** Autophagy, cellular degradation, chloroplast, photosynthesis, reactive oxygen species, signaling

## Abstract

Photosynthesis is an essential process that plants must regulate to survive in dynamic environments. Thus, chloroplasts (the sites of photosynthesis in plant and algae cells) use multiple signaling mechanisms to report their health to the cell. Such signals are poorly understood but often involve reactive oxygen species (ROS) produced from the photosynthetic light reactions. One ROS, singlet oxygen (^1^O_2_), can signal to initiate chloroplast degradation, but the cellular machinery involved in identifying and degrading damaged chloroplasts (*i.e*., chloroplast quality control pathways) is unknown. To provide mechanistic insight into these pathways, two recent studies have investigated degrading chloroplasts in the *Arabidopsis thaliana* ^1^O_2_ over-producing *plastid ferrochelatase two* (*fc2*) mutant. First, a structural analysis of degrading chloroplasts was performed with electron microscopy, which demonstrated that damaged chloroplasts can protrude into the central vacuole compartment with structures reminiscent of fission-type microautophagy. ^1^O_2_-stressed chloroplasts swelled before these interactions, which may be a mechanism for their selective degradation. Second, the roles of autophagosomes and canonical autophagy (macroautophagy) were shown to be dispensable for ^1^O_2_-initiated chloroplast degradation. Instead, putative fission-type microautophagy genes were induced by chloroplast ^1^O_2_. Here, we discuss how these studies implicate this poorly understood cellular degradation pathway in the dismantling of ^1^O_2_-damaged chloroplasts.

Plants use their chloroplasts as sensors of their environment. This is due to photosynthesis being sensitive to perturbation by environmental changes, which can lead to the production of reactive oxygen species (ROS) within chloroplasts.^1^ High levels of ROS, including singlet oxygen (^1^O_2_) and hydrogen peroxide (H_2_O_2_), can damage chloroplast structures and photosynthetic machinery, but can also signal for stress acclimation.^2^ For instance, accumulation of ^1^O_2_ in the chloroplast can signal to regulate chloroplast degradation, the expression of hundreds of nuclear-encoded stress- and photosynthesis-related genes, and eventual cell death.^3–5^ However, ^1^O_2_ is particularly reactive, has a short half-life (4 µsec) and diffusion distance (~220 nm), and is unlikely to leave the chloroplast (2–3 μm wide) in which it is generated.^6^ Thus, ^1^O_2_ likely leads to the local damage of chloroplast macromolecules, which may then act as secondary signals to promote these outcomes. H_2_O_2_ also has signaling capabilities, but it properties within a cell (a more stable half-life (1 ms), a longer diffusion distance (1 µm)) and ability to cross cellular membranes, allow it to exit organelles. Thus, it may be a less specific ROS for chloroplast stress.^7,8^ The mechanisms controlling ^1^O_2_-induced signaling and degradation are poorly understood,^9^ but the ability to degrade photo-damaged chloroplasts may provide a chloroplast quality control (CQC) system to ensure cells contain a healthy population of chloroplasts that perform efficient photosynthesis.

The *Arabidopsis thaliana plastid ferrochelatase two* (*fc2*) mutant, which accumulates chloroplast ^1^O_2_, has provided insight into ^1^O_2_-initiated CQC and cell death.^4^ The *fc2* mutation leads to the accumulation of the photo-sensitizing tetrapyrrole intermediate protoporphyrin-IX and a subsequent burst of ^1^O_2_ under diurnal light cycling conditions, causing wholesale chloroplast degradation and cell death in photosynthetic tissue. Under permissive constant light conditions, however, selective chloroplast degradation is observed, and individual chloroplasts are targeted for degradation in the cytoplasm. In some cases, these degrading chloroplasts protrude or “bleb” into the central vacuole, possibly for final turnover (Figure 1).^4^ These hallmarks of *fc2* mutant physiology make for an ideal system for genetic analyses to identify genes that play a role in ^1^O_2_-induced CQC and cell death (Figure 2a). So far, such genetic analyses have implicated chloroplast ubiquitination,^4^ the E3 ubiquitin ligase Plant U-Box 4 (PUB4),^4,10^ and plastid gene expression as playing roles in initiating ^1^O_2_-induced CQC.^11,12^ However, little is known about the cellular degradation machinery involved in recognizing and recycling ^1^O_2_-damaged chloroplasts.

**Figure 1.**
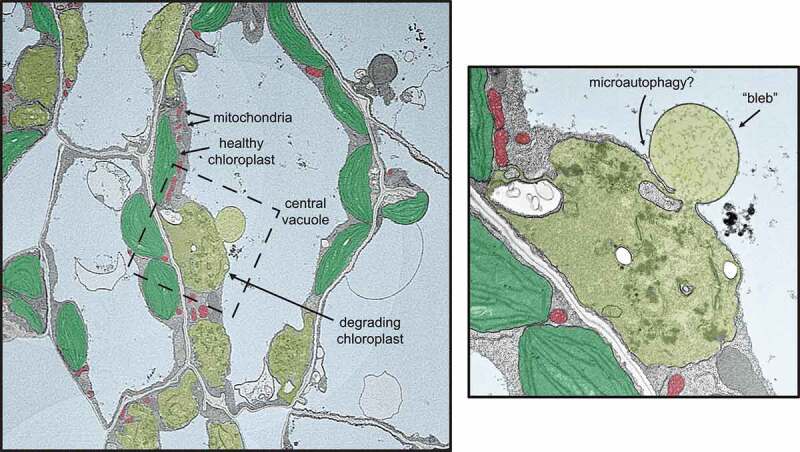
Singlet oxygen-induced selective chloroplast degradation.

False-colored transmission electron microscopy (TEM) micrograph showing the selective nature of ^1^O_2_-induced chloroplast degradation in the *Arabidopsis thaliana plastid ferrochelatase two* mutant. In the center, a “blebbing” event can be observed in which the degrading chloroplast (olive green) interacts with the central vacuole (light blue). Next to the degrading chloroplast are healthy chloroplasts (green) and mitochondria (red) in the same cell. A zoomed-in view of the bleb (right panel) highlights the similarities with fission-type microautophagy and endocytosis, where vacuolar membrane remodeling is “pulling” the blebbing chloroplast into the vacuole (as indicated by the close association of the chloroplast envelope with the vacuolar membrane (tonoplast)). Additionally, no double-membrane autophagosome structures are observed associating with chloroplasts.

**Figure 2.**
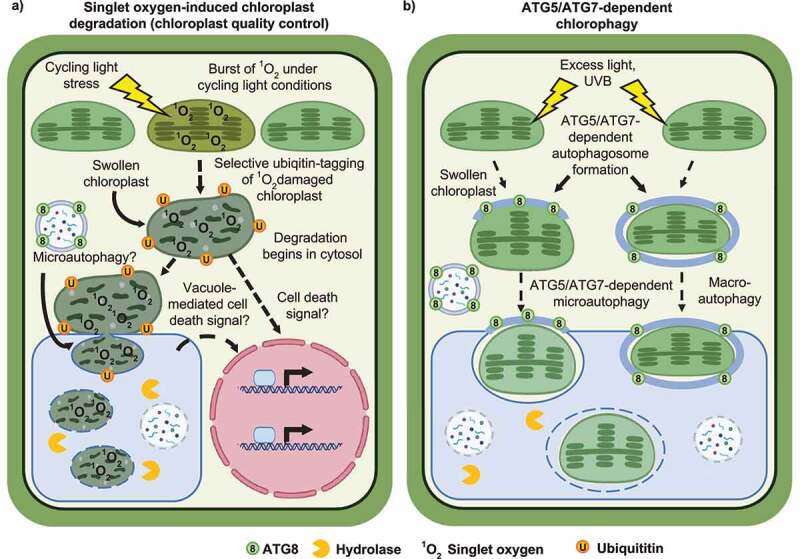
Models for chloroplast quality control pathways.

Hypothetical model contrasting ^1^O_2_-induced chloroplast quality control (CQC) (A) and chlorophagy (B). In the *plastid ferrochelatase two* mutant, ^1^O_2_-induced chloroplast degradation (A, left) proceeds by an autophagosome-independent process that resembles fission-type microautophagy. Under extreme ^1^O_2_ accumulation, degrading chloroplasts in the cytoplasm and/or vacuole may also signal to the nucleus to trigger a cell death pathway through an unknown signaling mechanism (A, right). Damaged chloroplasts swell after excess light exposure (B, left) and are transported into the central vacuole via autophagosome-dependent microautophagy. Alternatively, under UVB exposure, whole chloroplasts may be transported to the central vacuole via macroautophagy (B, right). In both ^1^O_2_-induced CQC and chlorophagy, chloroplast swelling correlates with their vacuolar transport. Figure created with BioRender.com.

Autophagy is a eukaryotic process that plays essential roles in cellular degradation, quality control (QC), and nutrient remobilization and can direct cell fate decisions, including senescence and cell death.^13^ This process can be used to turnover dysfunctional organelles as is the case in mitophagy, the autophagic transport of mitochondria to the vacuole (yeast/plants)/lysosome (animals).^14^ Canonical autophagy and autophagosome formation involve core autophagy (ATG) proteins in a ubiquitination-like mechanism that results in the tagging of cytosolic cargo with ATG8, a ubiquitin-like protein. Here, ATG7 acts like an E1 ubiquitin ligase, ATG10 acts like an E2 ubiquitin ligase, and ATG5 acts like an E3 ubiquitin ligase.^15^ Loss of any of these core ATG proteins (ATG5, ATG7, or ATG10) results in the loss of canonical autophagosome formation and autophagosome-dependent autophagy.^15–17^ In plants, autophagy can also be used to transport chloroplasts to the central vacuole. This can be in response to carbon starvation in the dark^18^ or in response to some types of photo-oxidative damage in a process called chlorophagy (Figure 2b). Chlorophagy can be induced by ultraviolet B-ray light (UVB)^19^ or excess light^20^ treatments, where photodamage leads to the transport of damaged chloroplasts to the central vacuole in a selective process that dependads on core autophagy-related (ATG) proteins, ATG5 and ATG7. This process occurs via canonical macroautophagy or autophagosome-dependent microautophagy after UVB or excess light stress, respectively (Figure 2b). Macroautophagy occurs via a well-characterized process that is generally dependent on core ATGs to direct cytosolic cargo transport to the vacuole/lysosome.^15^ ATG-dependent microautophagy involves partial autophagosome formation and “pushing” of target cargo into the vacuole/lysosome.^21^ Interestingly, excess light stress also leads to chloroplast swelling (Figure 2b), which may be a mechanism by which autophagosomes can recognize damaged chloroplasts.^20^ This raises intriguing questions regarding ^1^O_2_-induced CQC: How are ^1^O_2_-damaged chloroplasts recognized by the cell, and what structures are involved? -and- Is autophagosome formation necessary for the transport of ^1^O_2_-damaged chloroplasts to the central vacuole for degradation?

## ^1^O_2_-induced chloroplast quality control involves vacuolar structures associating with swelling chloroplasts

To better understand the structures involved in ^1^O_2_-induced CQC, we focused on chloroplast degradation in *fc2* mutants under permissive conditions without cell death.^22^ In these conditions, *fc2* mutants still accumulate low levels of protoporphyrin-IX and ^1^O_2_, which may be responsible for triggering CQC. Transmission electron microscopy (TEM) analysis again showed that some chloroplasts are selectively degraded while adjacent cellular structures appear normal^22^ (Figure 1). A small subset of these chloroplasts protrude (or “bleb”) into the central vacuole without the obvious association of double-membrane autophagosomes. Visually, such an interaction is reminiscent of ATG-independent (fission-type) microautophagy, where vacuolar membranes surround cytosolic cargo independent of autophagosome function.^21^ A 3D TEM analysis revealed that up to 35% of degrading chloroplasts (8% of all chloroplasts) in *fc2* mutants interact this way with the central vacuole.^22^ The structures within the central vacuole varied in size (an average of 9 µm^3^ (14% of the associated chloroplast) but were as large as 178 µm^3^ (202% size of associated chloroplast). Notably, the chloroplast-vacuole connection point was small (≤7 µm^2^), indicating why such interactions were rarely detected with traditional 2D images.

Next, we aimed to determine why some chloroplasts may be selected for degradation and vacuolar transport. Using a field emission scanning electron microscopy (FE-SEM) tile-scanned dataset of entire cotyledon cross-sections, we observed no significant correlation between chloroplast position and likelihood of degradation.^22^ Interestingly, chloroplasts in spongy mesophyll cells were slightly more likely to be degraded than those in palisade mesophyll cells. Furthermore, chloroplast and plastoglobule swelling were both shown to correlate with ^1^O_2_ signaling and precede chloroplast degradation. Genes encoding plastoglobule proteins involved in chloroplast disassembly during senescence were also upregulated in *fc2* mutants.^22^ Thus, we hypothesize that ^1^O_2_-induced swelling may be a possible mechanism for recognizing damaged chloroplasts (similar to chlorophagy^20^) and that ^1^O_2_-induced CQC may overlap with senescence pathways. Why damaged chloroplasts swell is unknown, but damaged mitochondria (also membrane-bound, energy producing organelles) swell due to the opening of nonselective channels and a loss of ion homeostasis.^23^ The possibility that chloroplasts swell due to a similar mechanism has not been fully explored.

### Investigating the role of core autophagy machinery in ^1^O_2_-induced CQC and cell death

As chlorophagy has been shown to occur by an ATG5- and ATG7-dependent process,^19,20^ we investigated if similar mechanisms are involved in ^1^O_2_-induced CQC. ATG-gene expression is activated in ^1^O_2_-stressed *fc2* mutants, but the resulting autophagosomes were not observed to associate with chloroplasts. Instead, autophagosomes only associated with *fc2* chloroplasts under carbon starvation (dark) conditions that should lack ^1^O_2_ accumulation.^24^ The role of autophagosome assembly in ^1^O_2_-induced CQC was then tested by introducing *atg5* and *atg7* null mutations into the *fc2* background. Neither mutation suppressed cell death or chloroplast degradation during ^1^O_2_ production.^24^ Importantly, chloroplast blebbing into the central vacuole was still observed in *fc2 atg* double mutants. These analyses make clear that such hallmarks of ^1^O_2_-induced chloroplast damage in *fc2* are not dependent on autophagosomes and, thus, is distinct from ATG5- and ATG7-dependent chlorophagy. This conclusion is supported by recent work showing that chlorophagy acts independently of PUB4^25^, which is involved in ^1^O_2_-induced CQC.^4^ Chlorophagy is also visually distinct from ^1^O_2_-induced CQC (chloroplasts remain relatively intact-looking even after being transported to the central vacuole^19^ (Figure 2b)) and requires at least 24 h to be initiated^20^ (^1^O_2_-induced CQC can be activated within 3 h^4^). Finally, under UVB stress, chlorophagy involves H_2_O_2_, rather than ^1^O_2_, accumulation.^19^ Therefore, chlorophagy and ^1^O_2_-induced CQC may be independent, but parallel pathways to recycle chloroplasts.

Based on these data, we hypothesized that an alternative form of degradation, possibly ATG-independent (fission-type) microautophagy, is involved in ^1^O_2_-induced CQC. ATG-independent microautophagy is not well characterized in plants, but in yeast, it involves a mechanism resembling endocytosis where the vacuolar membrane surrounds cytosolic cargo, independent of autophagosome formation.^21^ In ^1^O_2_-stressed *fc2* seedlings, several putative microautophagy-related genes (inferred from yeast homology^25^) were induced, suggesting this degradation pathway is being activated and could be responsible for ^1^O_2_-induced CQC.^24^ It will be compelling to investigate the necessity of this process in CQC to determine if similar ATG-independent microautophagy-related processes are conserved in plants. However, the possibility remains that ^1^O_2_-induced CQC may depend on an as-of-yet uncharacterized vacuolar transport mechanism.

The chloroplast is a central communication hub in maintaining plant energy levels and fitness in response to dynamic conditions. The presence of multiple selective chloroplast degradation pathways highlights the importance of chloroplast degradation in response to various stresses, each of which may cause different types of damage to the chloroplast. Selective chloroplast degradation likely serves at least two essential functions: nutrient redistribution and protection from toxic ROS accumulation. The growing field of ^1^O_2_-induced CQC, chlorophagy, and plant microautophagy holds a strong potential to further our understanding of the intricate mechanisms involved in plant stress biology. Such understanding will lead to valuable advances that aid in developing crops that have an increased yield and survive in ever more extreme growth environments.

## List of abbreviations

Singlet oxygen: ^1^O_2_, Chloroplast quality control: CQC, Reactive oxygen species: ROS, Autophagy-related: ATG
